# Acoustic Focusing of Protein Crystals for In-Line Monitoring and Up-Concentration during Serial Crystallography

**DOI:** 10.1021/acs.analchem.2c01701

**Published:** 2022-09-02

**Authors:** Björn Hammarström, Thomas J. Lane, Hazal Batili, Raymond Sierra, Martin Wiklund, Jonas A. Sellberg

**Affiliations:** † Department of Applied Physics, KTH Royal Institute of Technology, S-106 91 Stockholm, Sweden; ‡ Center for Free-Electron Laser Science, Deutsches Elektronen-Synchrotron DESY, Notkestrasse 85, 22607 Hamburg, Germany; § Linac Coherent Light Source, SLAC National Accelerator Laboratory, Menlo Park, California 94025, United States

## Abstract

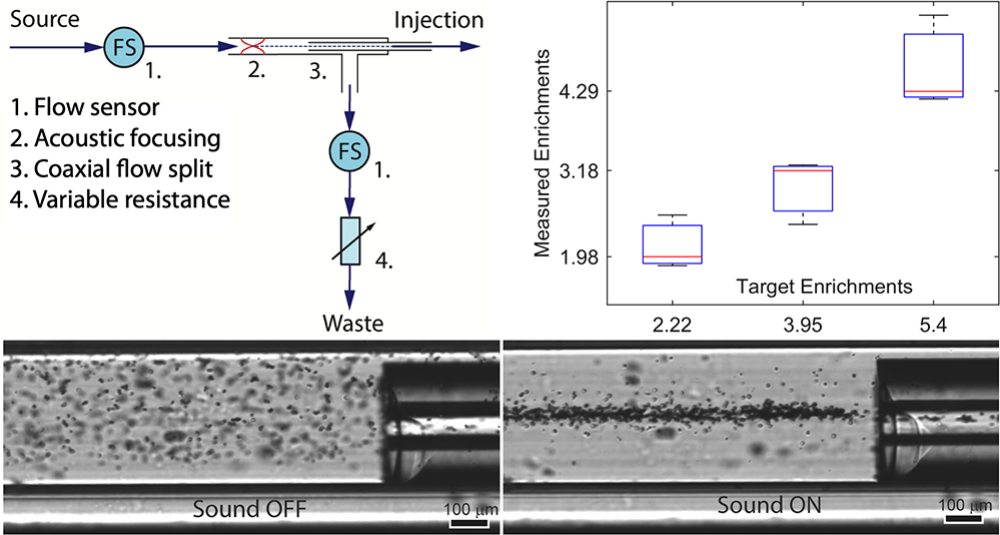

Serial femtosecond crystallography (SFX) has become one of the standard techniques at X-ray free-electron lasers (XFELs) to obtain high-resolution structural information from microcrystals of proteins. Nevertheless, reliable sample delivery is still often limiting data collection, as microcrystals can clog both field- and flow-focusing nozzles despite in-line filters. In this study, we developed acoustic 2D focusing of protein microcrystals in capillaries that enables real-time online characterization of crystal size and shape in the sample delivery line after the in-line filter. We used a piezoelectric actuator to create a standing wave perpendicular to the crystal flow, which focused lysozyme microcrystals into a single line inside a silica capillary so that they can be imaged using a high-speed camera. We characterized the acoustic contrast factor, focus size, and the coaxial flow lines and developed a splitting union that enables up-concentration to at least a factor of five. The focus size, flow rates, and geometry may enable an upper limit of up-concentration as high as 200 fold. The novel feedback and concentration control could be implemented for serial crystallography at synchrotrons with minor modifications. It will also aid the development of improved sample delivery systems that will increase SFX data collection rates at XFELs, with potential applications to many proteins that can only be purified and crystallized in small amounts.

## Introduction

The development of serial femtosecond crystallography (SFX) at X-ray free-electron lasers (XFELs) has pushed structural biology to unprecedented time resolution.^1−4^ It is based on delivering a continuously replenishing sample of protein crystals to the X-ray interaction region, where elastic scattering results in a diffraction pattern of the instantaneous structure prior to radiation damage according to the *diffraction before destruction* principle.^5,6^ By indexing individual diffraction patterns and merging the resulting still frames, a 3D structure of the electron density in the unit cell can be obtained with atomic resolution for radiation-sensitive photoenzymes^3,7^ or metal–organic chalcogenolates^8^ at room temperature.

Many approaches have been developed to reliably deliver protein crystals to the interaction region, including gas-dynamic virtual nozzles,^1,9^ double-flow focusing nozzles,^10,11^ lipidic cubic phase injectors,^12^ drop-on-demand injectors,^13,14^ high-speed fixed-target systems,^15^ and electrospun jets.^16,17^ Many of these nozzles were traditionally made by hand, but recent developments in 3D-printed nozzles^11,18^ have increased the reproducibility and feasibility to integrate microfluidics that enable, for example, chemical reaction initiation through mix-and-inject SFX^19^ or crystal bunching using optical traps.^20^ Despite significant development, sample delivery is still often the major bottleneck at SFX experiments. Here, we address some of these challenges, specifically those due to lack of in-line diagnostics, concentration control, and potential leaks and clogs at unions and nozzles.

Acoustic standing-wave manipulation of particles is a well-established technique to control cell growth, interaction, and selection (e.g., separation, trapping, and enrichment).^21−25^ The main advantage is that the acoustic radiation force is noninvasive and gentle compared to other external fields,^26,27^ enabling in-line focusing in capillaries and microfluidics without inducing cell damage. Another advantage is that the acoustic radiation force acts on any particle having an acoustic contrast relative its suspension medium, such as polystyrene beads,^28^ biological cells,^29^ bubbles,^30^ and also microcrystals.^31^ For sample alignment and injection purposes, acoustic manipulation has been used in flow cytometry,^32^ a technology that also has been commercialized (Attune flow cytometers, ThermoFisher Scientific, MA, US). Furthermore, acoustic forces are frequently used in drop-on-demand injectors^13,14^ and to trigger Rayleigh breakup,^9,33,34^ which results in uniformly sized and spaced droplets. However, few studies^31^ have investigated the use of the acoustic radiation force to focus protein crystals for SFX.

Herein, we use a piezoelectric actuator to create a standing wave perpendicular to the sample flow, focusing protein crystals into a single line inside a silicon microchip or a square silica capillary. This enables real-time online characterization of crystal size and shape in the sample delivery line after the in-line filter prior to injection. We characterize the acoustic contrast factor, focus size, and the coaxial flow lines in the capillary for the model protein lysozyme. Finally, as a proof-of-concept method, we investigate potential applications by developing a splitting union that enables up-concentration and discuss its impact for SFX of proteins that can only be purified and crystallized in small amounts.

## Methods and Devices

### Materials

Sodium chloride solution (5 M in H_2_O, BioReagent), sodium acetate trihydrate (BioUltra, ≥99.5%), lysozyme from chicken egg white (dialyzed, lyophilized, powder, ∼100 000 U/mg), acetic acid (BioUltra, ≥99.5%), and polyethylene glycol (PEG) 4000 were purchased from Sigma-Aldrich and used without further purification.

### Protein Crystallization

Lysozyme is a commonly used model protein for method development in X-ray crystallography. Therefore, lysozyme crystals were selected as a model for evaluating acoustic particle manipulation and to demonstrate the acoustically aided crystal injection method described herein. Lysozyme protein crystals were formed according to the protocol described below.

Lysozyme was prepared at 130 mg/mL in 50 mM sodium acetate, pH 3.5. This protein solution was mixed with a precipitant buffer of 1.25 M NaCl, 10% (w/v) PEG 4000, and 50 mM sodium acetate pH 3.5 to produce crystals. Both solutions were incubated at a set temperature (4, 12, 16, 25 °C); then, 300 μL of protein solution and 900 μL of precipitant were mixed and vortexed immediately. The mixed solution was incubated at the set temperature for 5–10 min, vortexed again, and then incubated at the set temperature overnight.

With this method, the temperatures of the protein solution and the precipitate when mixed will determine the final sizes of the protein crystals. For this study, crystals were formed at four temperatures (4, 12, 16, and 25 °C), producing crystals with approximate dimensions of 4, 8–11, 16, and 50 μm, respectively (Supporting Information).

### 1D Acoustic Focusing in a Silicon Microchip for Determining Acoustic Properties

Initial evaluations and determination of the acoustic contrast factor for the protein crystals were performed in a silicon microchip as this is a well-established platform for 1D acoustophoresis. The chip consisted of a 375 μm wide fluid channel, through-etched in a silicon wafer, and sandwiched between two layers of glass where access holes were drilled for the fluids. Actuation was provided by attaching a 1 mm thick lead zirconium titanate (Pb[Zr_*x*_Ti_1–*x*_]O_3_) piezoelectric transducer of grade PZT-4 (Pz26, Ferroperm, Denmark). Such a configuration provides 1D focusing of particles in a single node plane in the center of the channel when actuated by a frequency of 2 MHz as is well described in previous works.^35^

Determination of the acoustic contrast factor for the lysozyme crystals was performed comparing the speed at which they focused to that of polyamide beads (EU-DFS-BMF-ver.1 for Flow Doppler Phantoms, Danish Phantom Design, Denmark) with a known diameter of 5 μm and an acoustic contrast factor of  = 0.2386 in water.^35^ The comparison was performed at identical settings, albeit a different medium, and in the same imaging region of the microchannel. Particle tracking was done using TrackMate software.^36^

### Square Cross-Section Capillaries for 2D Acoustic Focusing

**Figure 1:**
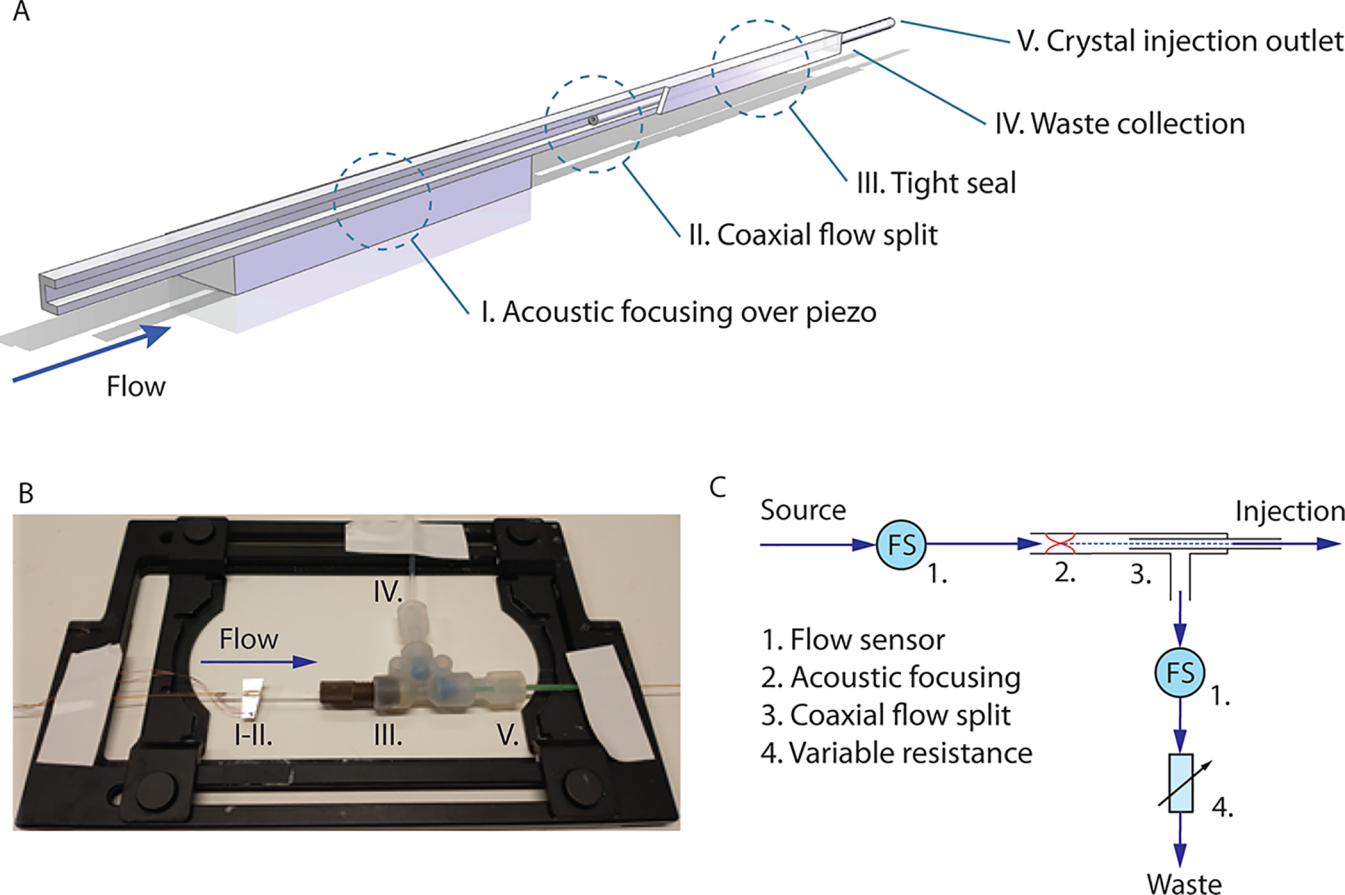
(A) Schematic setup for in-line enrichment of protein crystals. A square cross-section glass capillary is used to provide 2D acoustic focusing above a piezoelectric transducer. Collection of the central fluid fraction where the protein crystals are located is performed in a coaxial flow split. Such a flow split is accomplished by inserting a standard capillary into the square (acoustically focusing) capillary and directing the fraction going into the inserted capillary and the liquid around it to separate outlets. (B) Photograph of physical implementation of the enrichment device, which is straightforward and fits in a regular microscope holder where imaging can be done directly in the square capillary. Collection of the sample fractions directed toward the injection outlet and the waste outlet is accomplished by routing the capillary used for sample injection through a T-junction and into the square capillary. (C) Schematic of the fluidic setup for controlling the enrichment factor (i.e., the flow split ratio). Sample is fed through a sensor measuring the flow rate into the capillary and focused such that it follows the central stream into the injection capillary. A second flow sensor measures the fluid flow in the waste outlet, and a variable resistance tunes the split ratio between injection and waste outlets.

To integrate acoustic focusing with the glass capillary-based sample delivery systems used in SFX, it is desirable to provide acoustic focusing as close to the injection point as possible. Acoustic focusing in square cross-section capillaries was investigated as a route to achieve this (Figure 1A). Capillaries with inner diameters of 400 μm × 400 μm and wall thicknesses of 200 μm (VitroTubes 8240, VitroCom, NJ, US) were used, because a standard, cylindrical capillary with an outer diameter (OD) of 375 μm could be inserted directly. A 2 MHz piezoelectric actuator composed of lead zirconium titanate piezoelectric grade PZT-4 (Pz26, Ferroperm, Denmark) was attached to the square cross-section capillary using low-viscosity epoxy (EPO-TEK 301-1, Epoxy Technology, MA, US). 2D acoustic focusing of particles into a single line running in the center of the channel was provided by actuating the piezoelectric transducer using a linear frequency sweep from 1.87 to 1.97 MHz with an amplitude of 30 V_pp_. In addition, the square cross-section capillaries provided an improved ability to monitor the injection of protein crystals with microscopy as opposed to round capillaries.

### Coaxial Flow Splitter for Enrichment

To fully utilize the ability to acoustically focus particles in 2D, it is desirable to split the fluid streams in a coaxial fashion. This enables exclusive collection of the fluid fraction in the center of the channel to where the acoustic focusing directs the particles (Figure 1A).

Figure 1B shows the realization of such a union where an inlet capillary with an inner diameter (ID) of 100 μm is glued at the entry point of the square capillary. The protein crystals are subsequently focused by the ultrasound on top of the attached piezoelectric transducer. Downstream from this, a coaxial flow splitter is realized by routing the outlet capillary (crystal injection) through a large bore T-junction (IDEX HS, WA, US) and into the square capillary. By forming a tight seal around the square capillary at the T-junction port using a suitable sleeve and a ferrule with a metal collet (providing an even force to conform the sleeve to the square capillary), it was possible to direct the waste fraction away from the outlet and attach further fluidic components on that fluid line. Outlet capillaries with IDs of 100 and 20 μm were both evaluated in this study as they are commonly used for sample delivery.

The amount of enrichment is set by the split ratio between the fluid fractions going to the central outlet and the waste outlet on the side. This was controlled by implementing a system with two flow sensors (SLF3S-0600F, Sensirion, Switzerland) and a variable fluid resistance (Figure 1C). A solution with protein crystals was fed from a syringe pump (SP210C, World Precision Instruments, FL, US), and the flow rate (*Q*_in_) was measured using the first flow sensor. The flow rate at the waste outlet (*Q*_w_) was measured using a second flow sensor, and the ideal enrichment factor (*EF*^ideal^) was determined as
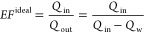
1

The flow rate in the waste line was controlled using a variable fluid resistance. Here, both pinch valves and attaching various lengths of capillary tubing were found to be effective means of tuning the split ratio. By designing the flow system in this fashion, the setup is not dependent on utilizing a high-pressure liquid chromatography (HPLC) pump or syringe pump for providing flow but could be adapted to various pressure sources, and the exit capillary is kept free from flow sensors and additional components.

## Theory and Modeling

### Determining Acoustic Contrast Factors by Particle Tracking

Particles exposed to a standing ultrasonic wave aligned along an axis (selected as *y*-axis in this study) will experience a primary acoustic radiation force (*F*_*y*_^rad^). As described by Bruus,^26^ the radiation force can be balanced to the viscous Stokes drag (*F*_*y*_^drag^) to produce a formula for the observed velocity of the particle
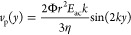
2where  is the acoustic contrast factor, *r* the radius of the particle, *E*_ac_ the acoustic energy density, *k* the wavenumber, and *η* the viscosity of the medium.

To determine the acoustic contrast factor of a particle experimentally by particle tracking, eq 2 was rearranged to the form
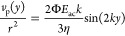
3

The left-hand side can now be determined for each particle by analyzing the *y*-velocity and the size of each individual particle–properties that can be extracted directly from a captured image sequence. Fitting a sine wave on the form, *A* sin(*b*(*y* – *c*)), to the measured values on the left-hand side of eq 3 allows a closer fit than fitting to the left-hand side of eq 2, since the size distribution of the particles is taken into account. With this fitting procedure, the properties of the background acoustic field are extracted using known particles by
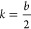
4
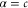
5
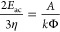
6where *α* represents a positional shift in *y* that aligns the nodal position with the measurements.

With known field properties, unknown particles can be investigated in a second step by reverse analysis
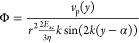
7where the *y*-velocity, *y*-position, and radius are properties extracted from the particle tracking.

As the particles approach their equilibrium positions (i.e., the pressure node in the case of positive contrast factors), the local concentration of particles will gradually increase. A dense particle suspension will lead to problems for most particle tracking algorithms and more pronounced effects of secondary radiation forces (i.e., acoustic particle–particle interactions). As has been suggested in previous publications,^35^ these challenges can be avoided by simply applying an exclusion region around the node. For the purpose of this study, a 30 μm wide exclusion band around the center of the channel was applied.

### Finite Element Model of the Cross-Sectional Resonances in a Square Capillary

**Figure 2:**
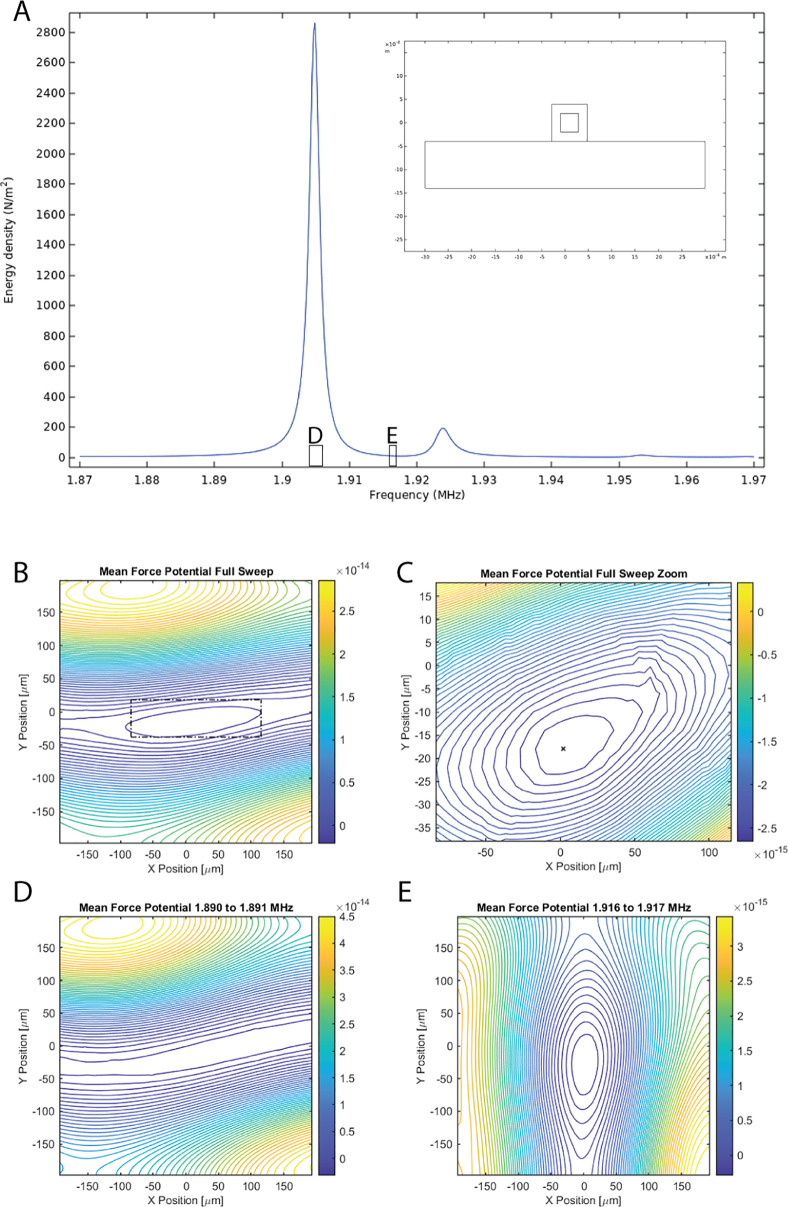
(A) Acoustic energy density from a 2D cross-section simulation of the square capillary with a constant voltage over the piezoelectric transducer for the frequencies in the applied sweep range. The full simulation geometry is shown as an inset, and the energy density is averaged over the part of the cross-section that is filled with water. Each frequency in this range will contribute to the final focusing but more so at the resonance peaks observed in the spectrum. (B) Integrated cross-sectional force potential in the full sweep range for a constant piezo voltage. (C) Close up of potential minima formed in the central region of the channel. In combination, (B) and (C) show that particles are expected to focus in 2D (both *x*- and *y*-directions) for a full range sweep. Particles will, however, focus at a faster rate in the *y*-direction (likely as it couples well to the piezo), but the focus position in the *y*-direction is also expected to be below the actual center of the capillary as the piezo asymmetrically adds material to the bottom of the capillary. (D, E) Sample potentials at shorter sweep intervals marked in (A). At such short intervals, 1D focusing is expected; this exemplifies how individual frequencies in the range will add up to the final 2D focusing in the capillary.

To model the acoustic fields used to create a 2D line focus in a square cross-sectioned capillary, a finite element simulation (Figure 2) was implemented in COMSOL (COMSOL Multiphysics v. 5.6, COMSOL AB, Stockholm, Sweden). A 2D cut-plane of the device (inset of Figure 2A) was placed in the region where the capillary was bonded to the piezoelectric transducer and modeled. The model utilized the piezoelectric interaction module for various frequencies, and the frequency range was chosen to match the sweep range used experimentally. The model used the built-in material parameters for water, borosilicate, and lead zirconium titanate (PZT-5H), and a fixed voltage over the piezoelectric transducer of 1 V was applied for each frequency. This allowed the pressure and velocity fields to be calculated for single-frequency actuation. From these fields, the acoustic energy density in the fluid channel and force potential for a polystyrene bead were calculated for each setting. The model is available for download in the Supporting Information.

Figure 2A shows the energy density in the fluid as a function of applied frequency using a fixed voltage. Two resonance peaks are visible in the spectrum, a main resonance between 1.90 and 1.91 MHz and a smaller peak between 1.92 and 1.93 MHz; at these peaks, a larger amount of acoustic energy is stored in the fluid when actuating with the same voltage.

When actuating using a fast frequency sweep, the resulting force potential will be the average of the potentials from the single frequencies in the range. In this situation, the patterns occurring at peaks in the energy density will have a larger contribution to the resulting force potential, but every frequency in the range will contribute. The average potential produced by a full frequency sweep from 1.87 to 1.97 MHz is shown in Figure 2B. The force potential displays a steeper gradient along the *y*-axis as the main peak in the energy density spectrum corresponds to a field aligned in this direction (Figure 2D). However, the frequency range also contains several frequencies where lateral focusing (i.e., *x*-direction) occurs, such as Figure 2E, and therefore, averaging the fields will enable focusing in two dimensions. This is obvious from Figure 2C, showing a close up of the central region of Figure 2B, where one can see a potential well with a minima located 15–20 μm below the center of the capillary in the *y*-direction and a few micrometers to the right of the center in the *x*-direction.

In summary, the simulations show the feasibility to achieve 2D focusing in a square capillary with a single transducer attached below the capillary if a frequency sweep is utilized. The focus is expected to be tighter in the *y*-direction due to the steeper gradient. One can also expect the focus to be slightly more off-centered in the *y*-direction than in the *x*-direction. This is most likely because the piezoelectric transducer adds material asymmetrically in the *y*-direction, which would extend the propagation distance for a standing wave in that direction. The asymmetry in the *x*-direction is caused by shifting the capillary 100 μm off center to avoid simulation artifacts that can arise from perfect symmetry. In reality, it is not possible to achieve a perfect symmetry in the *x*-direction, which means that asymmetry is often a necessity that can be exploited.^37^ It is worth noting that the simulations were performed with a PZT material with lower quality factor (*Q*-value) than the experimental device and that efficient 2D focusing may benefit from designing an acoustic manipulation system having a limited *Q*-value of the resonance. The reason is that a slightly lossy system will result in an acoustic energy spectrum with broader peaks^38^ (cf. Figure 6 in ref (38)). For example, two single-frequency resonances, each creating different force potentials, may only be excited simultaneously by choosing a driving frequency where the peaks overlap. We prefer to sweep the driving frequency over all useful resonance peaks in the energy density spectrum, since this is insensitive to small differences in force potentials due to capillary manufacturing or device assembly. Another interesting option is to exploit asymmetric resonator designs for improved performance in acoustic manipulation systems. Asymmetry is achieved in our system primarily by the piezoelectric transducer that is attached to one of the four sides of the square-shaped capillary (Figure 1A).

### Flow Simulation of Coaxial Flow-Splitting Capillary Union

**Figure 3:**
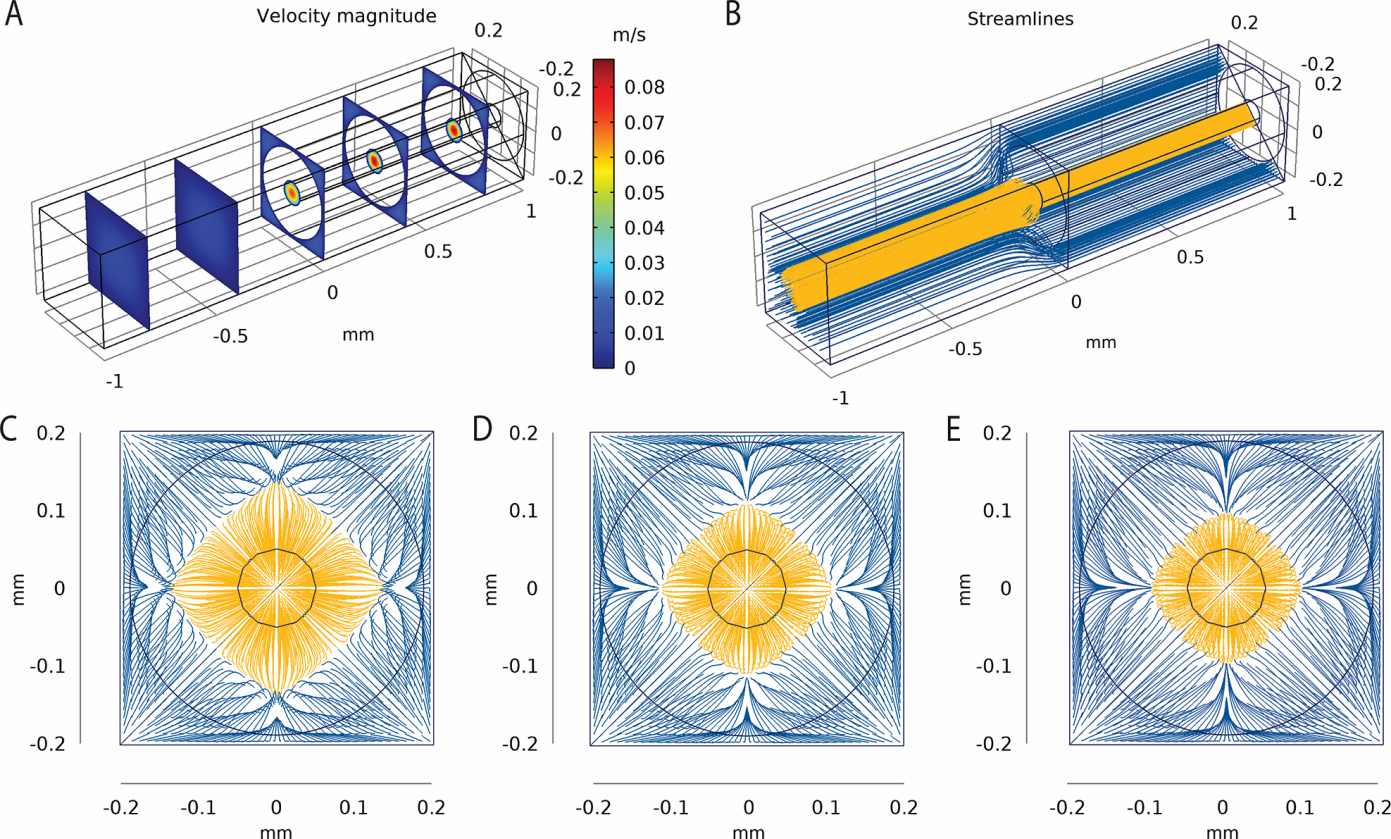
(A) Simulated flow velocity in the proposed device for coaxial flow splitting. (B) Color-coded streamlines linked to end points either in the core (yellow) or shell (blue) show the possible trajectories for either injection into the capillary or direction toward the waste. Viewing the streamlines from the point of entry (C–E) illustrates how tight the acoustic focus needs to be for directing particles to the core outlet. Split ratios of 1:1 (C), 1:2 (D), and 1:3 (E) are visualized, corresponding to 2, 3, and 4 times up-concentration if all particles are located in the yellow region. It may be noted that the yellow collection region is diamond shaped, reflecting the square shape of the outer capillary. In addition, the demands on acoustic focusing are not influenced by the bore of the central capillary as it is solely determined by the split ratio between the core and the shell streams.

A COMSOL simulation was implemented to simulate the fluid flow in the capillary union, used to create a coaxial flow split for extracting the central fluid fraction during enrichment. The simulation is shown in Figure 3 and was implemented in 3D using the laminar flow model at steady state and is available in the Supporting Information. The model simulates the fluid-filled regions in the geometry with one inlet and two outlets, one outlet at the bore of the inserted capillary, and one outlet in the region defined by the difference between the square capillary and the exterior of the round capillary. The flow rates were set such that the central-bore outlet had a flow rate of 20 μL/min (a typical injection rate used in SFX), the inlet a multiple of this flow rate, and the side-outlet left to be determined by the simulation (average pressure condition). Three different inlet flow rates were investigated, 40, 60, and 80 μL/min, corresponding to target enrichment factors (*EF*^ideal^) of 2, 3, and 4, respectively.

## Experimental Results and Discussion

### Acoustic Focusing of Protein Crystals

**Figure 4:**
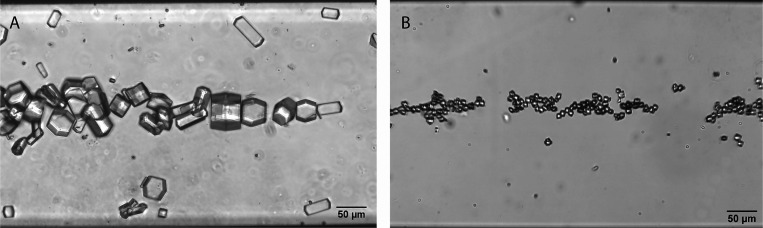
Lysozyme crystals generated at (A) 25 °C (∼50 μm) and (B) 12 °C (∼11 μm) are focused into a single line in a 2 MHz silicon microchannel. Videos of the 12 °C crystals going from a homogeneous distribution into a single line were used to evaluate the acoustic properties of the protein crystals.

When subjecting a solution of lysozyme protein crystals in their native medium to a standing ultrasonic wave, it was found that the protein crystals had a positive contrast factor and moved to the pressure nodes of the standing wave. This is demonstrated in Figure 4, where acoustic focusing of large-sized (50 μm) and medium-sized (11 μm) crystals are shown. Here, a microfluidic chip consisting of a 375 μm wide channel operated at 2 MHz was used to form a single nodal line in the center of the channel to which the protein crystals are attracted (videos of large-sized crystals and medium-sized crystals are available in the Supporting Information).

In contrast to the cells and polymer beads traditionally focused using acoustophoresis, the protein crystals are not round or approximately spherical. The shapes of the crystals are easily seen in the case of the large crystals (25 °C), but similar morphologies are present in the smaller crystals. For focusing of the crystals using primary radiation forces, the nonspherical particle shape did not seem to alter the expected behavior, although in some cases rotation of the particles was observed. It is expected that the fluidic drag of faceted crystals will be different than of spherical objects and depend on how the particle is aligned with respect to the direction of the acoustically induced travel. In future studies, crystalline particles might become useful as a controlled means for experimentally investigating how acoustic waves interact with nonspherical objects, but for the application at hand, we have focused on finding a metric for how easily the particles are focused using ultrasound. Therefore, the theory developed for round particles was applied, using a radius gained from the projected area of the crystal in each image to determine an *effective* acoustic contrast factor () that can be compared to other particles. As described in the Theory and Modeling section, the fluidic drag also comes into this calculation. An increase in fluidic drag due to the nonspherical shape would reduce the *effective* contrast factor.

**Figure 5:**
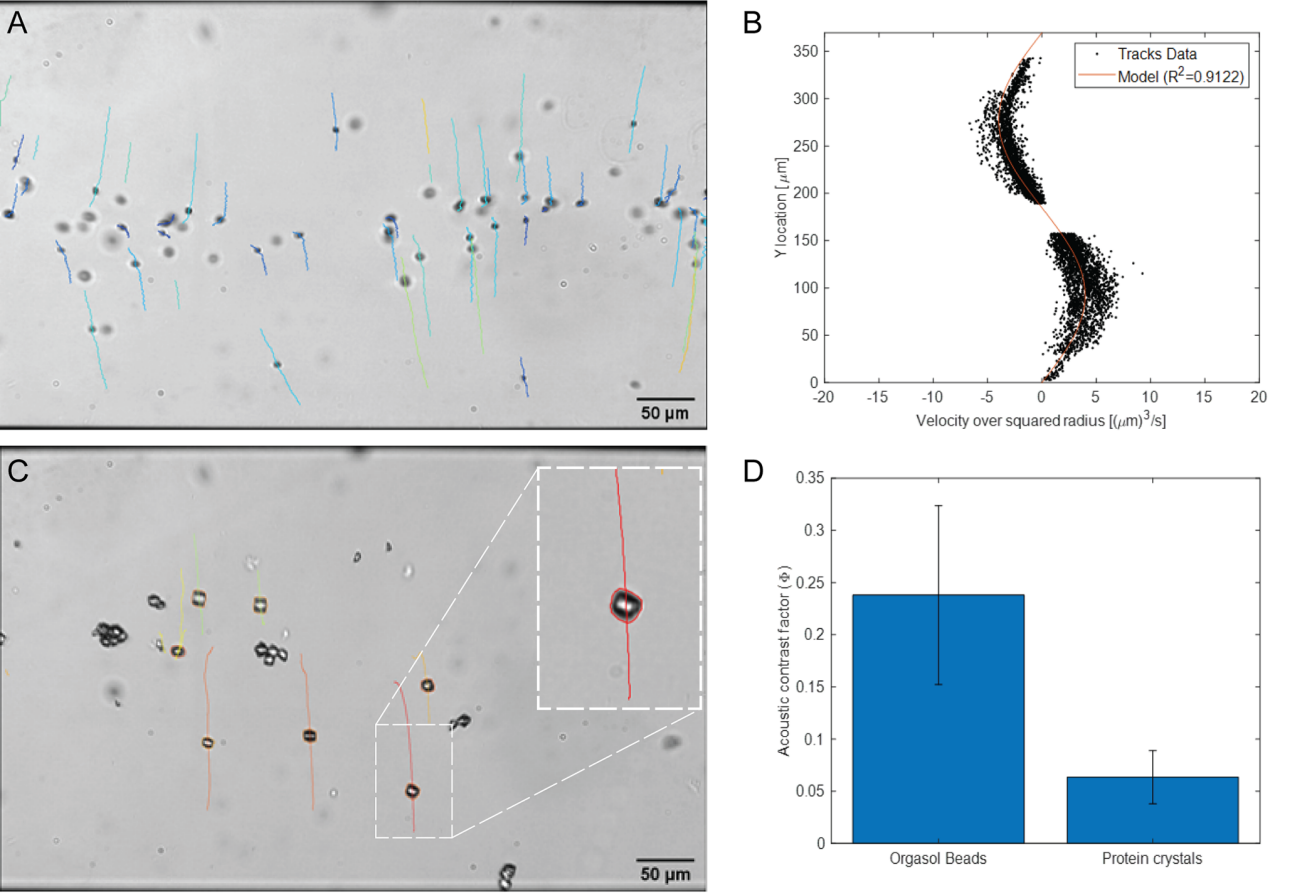
(A) Tracked calibration beads (polyamide) in water with known acoustic properties are used to establish the acoustic energy density in the channel. (B) By fitting a sine wave (R^2^ = 0.91) to the measured velocity divided by the individually measured particle radiuses, the acoustic energy density in the channel of 2.62 J/m^3^ is extracted (Supporting Information). As particles reach the center of the channel, they will come in close proximity of each other, which may create problems with the particle tracking algorithm as well as induce effects of acoustic particle–particle interactions. For this reason, an exclusion region (i.e., region where particles are not analyzed) of 30 μm in the center of the channel is used to enable tracking of single particles alone. (C) Trajectories and sizes of the lysozyme protein crystals are measured at the same ultrasound exposure and used to measure the acoustic contrast factor of the protein crystals in their native medium. (D) Comparison of the measured contrast factor for the calibration beads ( = 0.238 ± 0.086) to the protein crystals ( = 0.064 ± 0.025), showing that the protein crystals in their native medium will move as regular polystyrene beads in water but at a slower rate given the same particle diameter.

The contrast factors of the lysozyme crystals were determined by comparing the speed at which the crystals focused to the speed of polymer calibration particles with known average contrast factors (same settings and imaging region). Figure 5A shows polymer beads in water with particle tracks as an overlay, and Figure 5B shows the sinusoidal velocity distribution in the (vertical) *y*-direction. By normalizing each velocity track with the squared radius of each particle (*v/r*^*2*^), a much better fit (R^2^ = 0.9122) to the theoretical model is accomplished than if using a global value for the particle radius. Figure 5C shows a sample of protein crystals in their native medium with particle tracks in overlay (analyzing only isolated, single particles). It is worth noting that the energy density of 2.62 J/m^3^ in the channel during calibration will change when focusing is performed in crystal media instead of water. Comparing the measured *v/r*^*2*^ value for each particle and position to the model plotted in Figure 5B allows a contrast factor to be calculated for each particle despite changes in energy density. Figure 5D shows the results of this analysis where the calibration particles are also included to validate the model and to provide a comparison to the protein crystals.

In conclusion, the *effective* acoustic contrast of the lysozyme crystals is approximately 27% of the polymer particles making them move relatively slowly in an acoustic field. This implies that higher acoustic powers or lower flow rates are required to achieve equal focusing. When doing this comparison to previous works concerning cells,^27^ it is worth considering that an increased power, and in turn temperature, may not be as damaging to the protein crystals as it would be to a living cell. One reason for the relatively low acoustic contrast may be the shape of the crystals, which may lower the *effective* contrast due to an increased fluidic drag. Another reason may be that the acoustic contrast factor is determined by the acoustic properties of the particle in relation to the medium. For this study, we use the native medium in which the proteins are formed to impose minimal changes to current systems and diffraction quality of the crystals. However, native media may naturally have similar properties to the crystals and may therefore be a poor choice for providing a high acoustic contrast. For cases when buffer exchange is possible without altering the diffraction quality of crystals, it would be interesting to investigate whether it could provide a general improvement in acoustic manipulation.

### 2D Focusing of Protein Crystals in Capillary Unions

**Figure 6:**
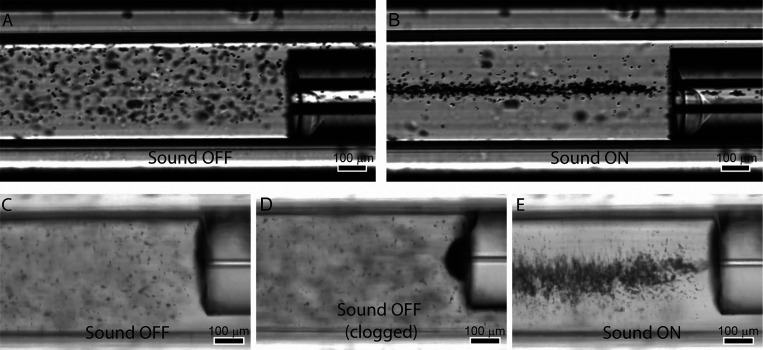
Injection with (A) ultrasound off and (B) ultrasound on for a 100 μm capillary union with 11 μm protein crystals. Injection with (C, D) ultrasound off and (E) ultrasound on for a 20 μm capillary union with 4 μm protein crystals. Clogs randomly occurring during injection without acoustic focusing (D). Fluid flow is from left to right in the images.

It was possible to focus lysozyme protein crystals formed under various conditions using a quadratic cross-section capillary. Figure 6 shows acoustic focusing of protein crystals in a 400 μm × 400 μm capillary at 20 μL/min, where panels A and B show 12 °C crystals (∼11 μm) and panels C–E 4 °C crystals (∼4 μm). It is also demonstrated that the quadratic cross-section capillary can be connected to capillary tubing of various inner diameters, 100 μm ID capillary for the large crystals and 20 μm ID capillary for the small crystals, as long as the OD is less than 400 μm.

This approach can be utilized to form capillary unions where crystals can be focused and imaged during injection. As opposed to a round capillary, the square capillary provides good conditions for imaging the crystals as they are injected into the smaller capillary. In a conventional capillary union, the ends of two capillaries are pressed against each other in a manifold. To prevent clogging of such a union, it is important to have precise cuts at the capillary ends and to mount them perfectly flush. If some distance separates the capillaries, there will be a region with a significantly wider cross-section (i.e., dead volume). A widened cross-section will reduce the particle velocity and amplify any issues with sedimentation or aggregation that can lead to buildup of particles and eventual clogging of the capillary. Such a scenario is shown in Figure 6D where the sound was deactivated for a longer time period leading to eventual clogging of the capillary. In addition, transitioning from a larger to a smaller capillary can also be problematic as flow lines cannot conform to the sudden decrease in cross-section and will leave a region of stagnant flow where a potential buildup of particles can occur. By separating the capillaries by a significant distance and using acoustics to lift and focus the particles before injection to the downstream capillary, the injection process can be monitored using optical imaging. Furthermore, there are no requirements on mounting the capillaries perfectly flush or limitations on which capillary dimensions must be matched.

**Figure 7:**
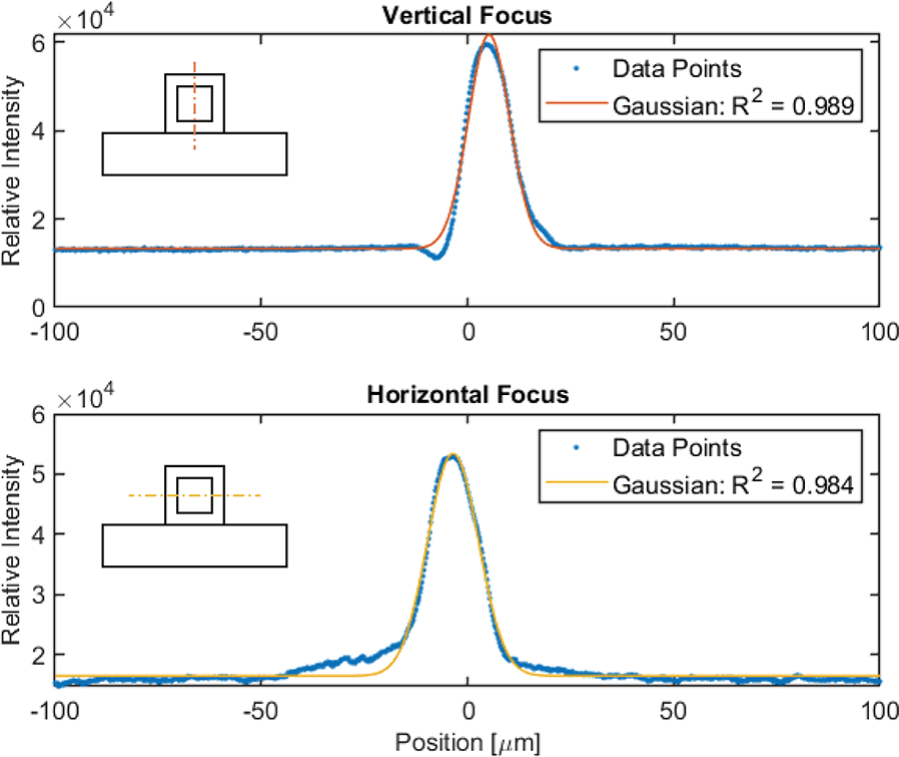
Integrated intensities for image sequences taken from the side (vertical) and the top (horizontal) of the capillary while running 8.5 μm-sized lysozyme crystals at 40 μL/min. The side sequence has its maximum 5.22 μm from center (μ) and a deviation (σ) of 5.02 μm. The top sequence has its maximum 3.52 μm from center (μ) and a deviation (σ) of 6.18 μm. As in the force simulations, the acoustic focus is tighter in the vertical direction but more offset from the center (due to the added material of the piezoelectric ceramic). Focusing in the horizontal direction is also achieved, although coupling to the piezoelectric transducer is weaker in this direction.

2D focusing of particles (both *x*- and *y*-direction) is crucial for operation of the device. This was achieved within less than 15 μm (fwhm) by utilizing a frequency sweep from 1.87 to 1.97 MHz in accordance with the simulations. As the capillary could be imaged both from the top and the side, by turning it 90°, the 2D focusing could be evaluated experimentally. By imaging a section of the capillary with focused protein crystals flowing at 40 μL/min and integrating the image intensity over time, the particle distribution could be determined, as shown in Figure 7. This showed successful focusing of particles in both directions.

As predicted by the simulations, there will be a stronger force in the vertical direction as the piezoelectric actuator is positioned to couple directly into this oscillation, which is consistent with the tighter vertical focus (σ = 5.02 μm). The offset from the center of the capillary is also larger in the vertical direction, which can be seen in the simulations. This could be attributed to the fact that the piezoelectric transducer itself will add material to one side and thereby push the node slightly off-center in the vertical direction.

### Enrichment-Enabled Capillary Union

**Figure 8:**
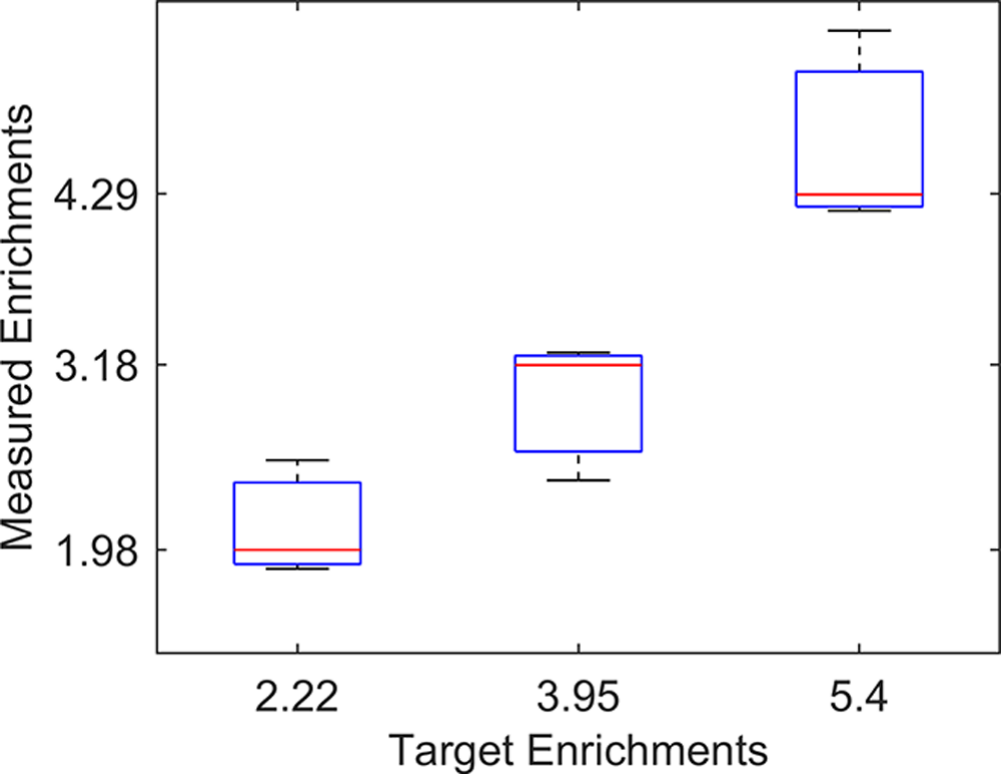
In-line enrichment of lysozyme crystals is determined by the split ratio between liquid going into the capillary and liquid passed on to the sides. It was possible to reliably get over four times enrichment, and the highest enrichment measured was 5.3 times the initial concentration. For all experiments, a constant injection flow rate of 20 μL/min was used to simulate normal operating conditions for crystallography, meaning that protein crystals are focused at 108 μL/min in order to perform enrichment at the highest setting. Visual observations showed that the limiting factor in the experiments was not the ability to focus the crystals but were alignment of the focused line of particles and the coaxial flow-splitting union. Higher enrichment rates could be possible by refined methods for aligning the core capillary in the flow-splitting union or by reducing the injection flow rate in the exit capillary below 20 μL/min.

In combination with coaxial flow split, the 2D acoustic focusing allows in-line enrichment of protein crystals directly prior to injection. The ideal enrichment factor, at 100% particle recovery, is compared to the experimental enrichment factor in Figure 8. It was possible to reliably achieve an enrichment factor above four times the original concentration. This was done while maintaining a flow rate of 20 μL/min at the injection port, which corresponds to 2D acoustic focusing of particles at 108 μL/min in the main capillary. The resulting enrichment factors are useful as a means to fine-tune and optimize the hit-rate during an experiment and to compensate for the gradual decrease in particle concentration that is typically observed during longer experiments (caused by, for example, sedimentation in the reservoir).

In this study, an exit flow of 20 μL/min was used to simulate relevant flows in a 100 μm ID exit capillary. This flow rate is typically not required at the injection nozzle but is needed to achieve a practically useful particle/crystal velocity during transport to the nozzle. If, however, a smaller ID capillary is used for sample delivery to the nozzle, lower flow rates could allow significantly higher enrichment factors.

As seen in Figure 8, the enrichment level does not plateau above the maximally achieved enrichment. Instead, it becomes unstable due to limitations in alignment between the focused particle stream and the flow-splitting union. At target enrichments above the presented settings, a focused stream of particles directed at the edge of the exit capillary but breaking off into the side outlet was observed. This suggests that it is not how narrow the acoustic focus is that is the limiting factor, but rather the alignment between the acoustic focusing line and the central selection region of the coaxial flow split. In the current setup, two main sources of misalignment can be identified. First, as seen experimentally and in simulations, the acoustic focus is not perfectly centered in the quadratic capillary. Second, the OD of the inserted capillary is specified to 375 μm, while the quadratic capillary has a side length of 400 μm. In comparison, the offset in acoustic focusing was experimentally characterized to the range of 4–5 μm, while the coaxial union could account for a 12.5 μm offset in a worst-case scenario. With a more advanced manifold for aligning the acoustic focus with the central collection region in the coaxial flow split, it is likely that significant improvements to the enrichment factors could be made.

**Figure 9:**
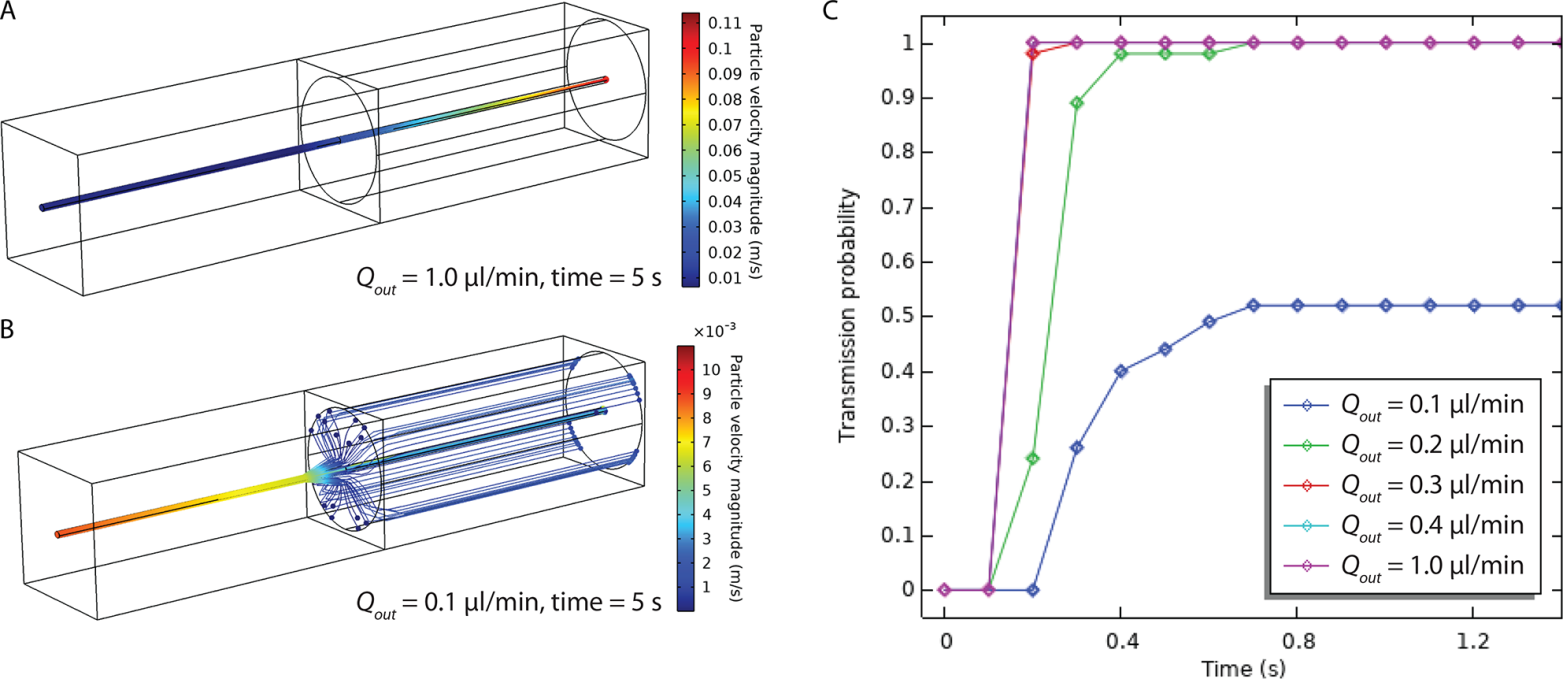
Particle tracing simulation of focusing at 40 μL/min and injection to a small-bore capillary (20 μm) aligned with the acoustic focus. (A) With an outflow of 1 μL/min, all particles entering the system within the acoustic focus go to the center outlet, and (B) with an outflow of 0.1 μL/min, half of the particles go to the center. (C) The transmission probability (acoustic focus to central outlet) goes to 1 for outflows above 0.2 μL/min. With this outflow and the acoustic focus presented herein, it is therefore possible to achieve enrichment factors of around 200 with optimal alignment.

To project a maximal up-concentration with the acoustic focusing demonstrated in Figure 7, a flow simulation was conducted to show the effect of optimized alignment and exit-flow. In this simulation the in-flow was fixed at 40 μL/min (corresponding to the experimental setting), and the exit-flow was varied in the range between 1 and 0.1 μL/min. Particles were released from an ellipse with a major axis of 12.36 μm and a minor axis of 10.04 μm (4σ) corresponding to the region where 95.4% of all particles were located experimentally. The result of the simulation is shown in Figure 9, where full recovery of the particles is obtained at 0.2 μL/min exit-flow, and approximately 50% recovery is obtained at 0.1 μL/min. With an injection rate of 0.2 μL/min, it is therefore possible to obtain an enrichment factor of 200 with close to complete recovery—provided an optimal alignment of the stream of focused particles and the coaxial flow splitter. For an experiment requiring an injection rate of 1 μL/min, the enrichment factor would be 40. Here, the simulations show that the acoustic focus is more than what is required, such that there is potential for further up-concentration by instead increasing the in-flow.

## Conclusion

We have investigated the possibility of using a piezoelectric actuator to focus lysozyme crystals with dimensions ranging from 4 to 50 μm using the acoustic radiation force. An effective acoustic contrast of 0.064 ± 0.025 was established in the native buffer, which could likely be increased by buffer exchange to a medium with larger density difference with respect to the protein crystals. 2D acoustic focusing was achieved within 15 μm (fwhm) at 40 μL/min in a square capillary with a frequency sweep from 1.87 to 1.97 MHz. As a proof-of-concept method, a coaxial flow-splitting union enabled in-line enrichment factors up to at least five, limited by the alignment of the acoustic focus with the central collection region in the coaxial flow split. By optimizing the alignment and reducing the output flow rate to 0.2 μL/min, an up-concentration as high as 200 fold may be achieved. In-line diagnostics using 2D acoustophoresis could be implemented at a synchrotron beamline to perform serial crystallography with improved hit rates directly within the square capillary using a 1D X-ray focus. It could also be combined with 3D-printed nozzles in a single device to enable concentration control at XFELs and increase SFX data collection rates, which would widen the potential applications to proteins that can only be purified and crystallized in small amounts.

## Supporting Information Available

Additional experimental and simulation details (PDF)
Acoustic model (ZIP)
Fluid flow model (ZIP)
Particle tracing and fluid flow model (ZIP)
Video of acoustic focusing of large crystals (ZIP)
Video of acoustic focusing of concentrated medium-sized crystals (ZIP)
Video of acoustic focusing of diluted medium-sized crystals (AVI)

## Supplementary Material

ac2c01701_si_006.zip

ac2c01701_si_007.avi
