# Supplementary material for: Acoustic Focusing of Protein Crystals for In-Line Monitoring and Up-Concentration during Serial Crystallography
Source: Anal Chem. 2022 Sep 2;94(37):12645–56. doi: 10.1021/acs.analchem.2c01701 (PMC9494305; doi:10.1021/acs.analchem.2c01701)
Supplement: Supplementary file 1 — ac2c01701_si_001.pdf [file ac2c01701_si_001.pdf]

Supporting Information

Acoustic focusing of protein crystals for in-line monitoring and up-concentration during serial crystallography

Björn Hammarström[1*], Thomas J. Lane[2], Hazal Batili[1], Raymond Sierra[3], Martin Wiklund[1] and Jonas A. Sellberg[1*]

1 Department of Applied Physics, KTH Royal Institute of Technology, S-106 91 Stockholm, Sweden;

2 Center for Free-Electron Laser Science, Deutsches Elektronen-Synchrotron DESY, Notkestrasse 85, 22607 Hamburg, Germany;

3 Linac Coherent Light Source, SLAC National Accelerator Laboratory, Menlo Park, California 94025, USA;

** Authors to whom correspondence should be addressed: bham@kth.se; jonassel@kth.se*

Table of Contents

Supporting Figures **S2**
Approximation of protein sizes *S2*
Attraction of crystals to sharp edges *S2*
Energy density and impedance for the finite element model *S3*

Supporting Texts **S4**
Energy density for calibration measurements *S4*

Supporting Files **S5**
Acoustic model *S5*
Fluid flow model *S5*
Particle tracing and fluid flow model *S5*
Video of acoustic focusing of large crystals *S5*
Video of acoustic focusing of concentrated medium-sized crystals *S5*
Compressed video of acoustic focusing of diluted medium-sized crystals *S5*

Supporting Figures

Approximation of protein sizes

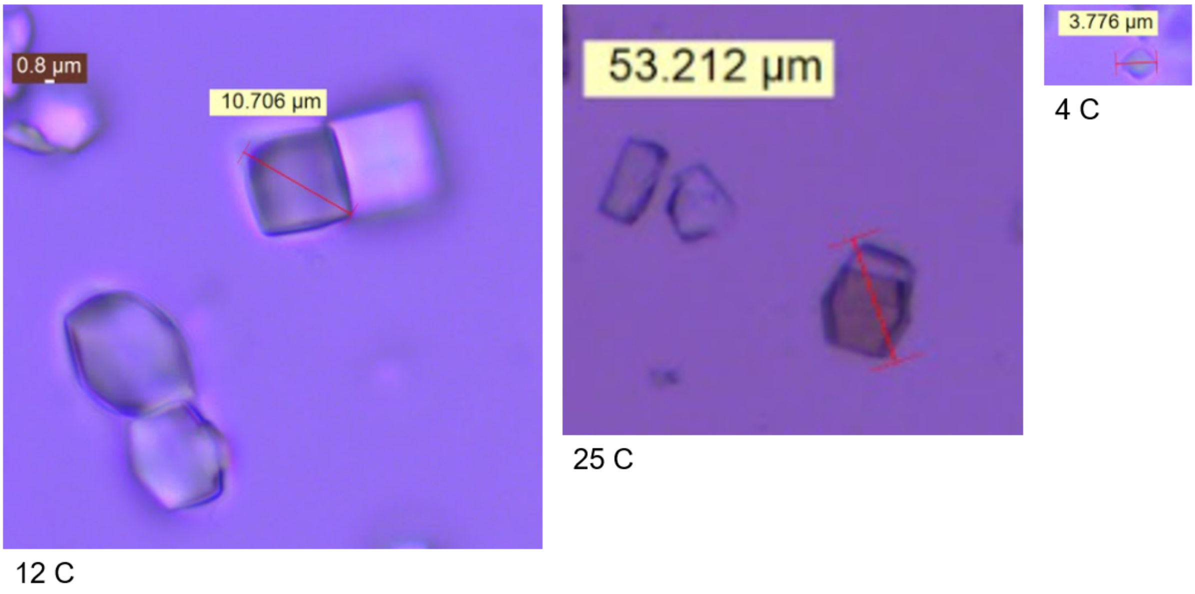

Figure S1. Approximate sizes of protein crystals formed at 12, 25 and 4ºC.

Attraction of crystals to sharp edges

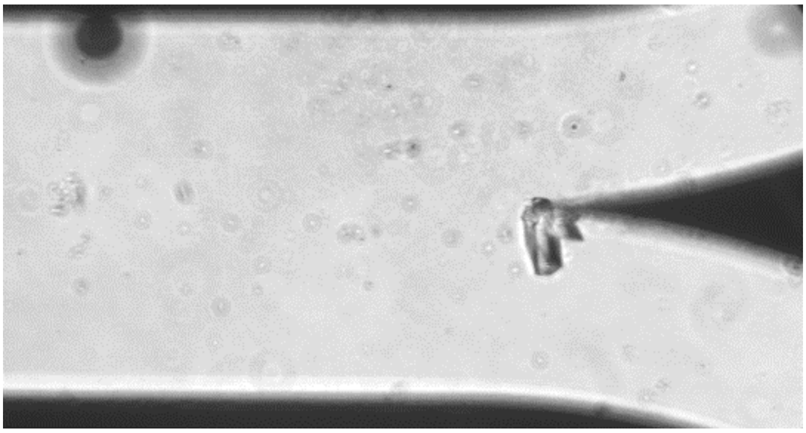

Figure S2. The crystals have a tendency to be attracted and trapped by sharp edges under flow in silicon microchannels, which could be utilized to automatically mount single crystals of certain sizes at crystallography beam lines.

Energy density and impedance for the finite element model

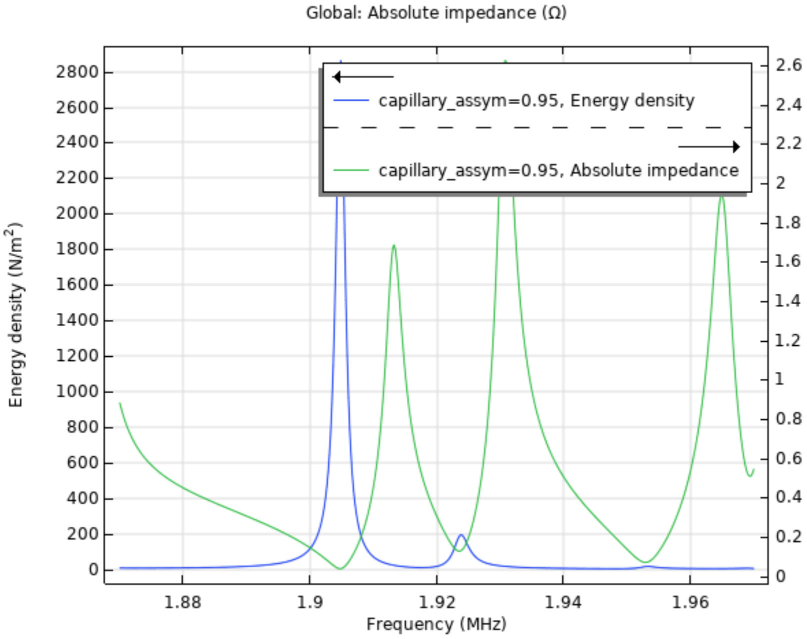

Figure S3. Energy density (blue) and absolute impedance (green) for the finite element model of the cross-sectional resonances in a square capillary (see Figure 2); 'capillary_assym' is an optional parameter for evaluating the impact of differences in *x*- and *y*-dimensions.

Supporting Texts

Energy density for calibration measurements

To derive the energy density, E_{den}, from a tracked particle with known properties the following analysis can be used. Here, the velocity, v, and the radius, r, are measured, whereas ϕ, is the acoustic contrast factor and the dynamic viscosity, η, are known constants. A, k, and φ, represent the amplitude, wavenumber and the phase of a sine wave fitted to measurements from all tracked particles.

$$v = \frac{2}{3}\phi \mathrm{k} r^2 E_{\text{den}} \frac{1}{\eta} \sin\left(2\mathrm{kx} - \varphi\right) \tag{S1}$$

$$\frac{v}{r^2} = \frac{2}{3}\phi k E_{\text{den}} \frac{1}{\eta} \sin\left(2\mathrm{kx} - \varphi\right) \tag{S2}$$

$$A = \frac{2\phi k}{3\eta} E_{\text{den}} \Rightarrow E_{\text{den}} = \frac{3A\eta}{2\phi k} \tag{S3}$$

Using the calibration particle data and acoustic contrast in the main paper (with η = 0.890 mPas), the energy density during tracking was estimated to 2.62 J/m^3 for beads in water.

Supporting Files

Acoustic model
Simulates resonances in the quadratic cross-section capillary and relates to Figure 2 and Figure S3.
Filename: **sqcap_on_pzt_sup.mph**

Fluid flow model
Simulates flow in the coaxial union and relates to Figure 3.
Filename: **flow_in_union_1_sup.mph**

Particle tracing and fluid flow model
Simulates flow and traces particles in the coaxial union with the measured acoustic focus and relates to Figure 9.
Filename: **flow_in_union_2_ptrack_sup.mph**

Video of acoustic focusing of large crystals
Shows how acoustophoresis can be applied to focus large (50-µm) lysozyme crystals.
Filename: **sichip_xtals_25c_r4.gif**

Video of acoustic focusing of concentrated medium-sized crystals
Shows how acoustophoresis can be applied to focus medium-sized (11-µm) lysozyme crystals at high concentration.
Filename: **sichip_xtals_12c_high_density.gif**

Compressed video of acoustic focusing of diluted medium-sized crystals
Shows how acoustophoresis can be applied to focus medium-sized (11-µm) lysozyme crystals at low concentration. Uncompressed video available upon request.
Filename: **sichip_xtal_12c_dil_r1.avi**
